# Addressing relationship quality of people with dementia and their family carers: which profiles require most support?

**DOI:** 10.3389/fpsyt.2024.1394665

**Published:** 2024-09-11

**Authors:** Maria J. Marques, Bob Woods, Hannah Jelley, Liselot Kerpershoek, Louise Hopper, Kate Irving, Anja Bieber, Astrid Stephan, Anders Sköldunger, Britt‐Marie Sjölund, Geir Selbaek, Janne Røsvik, Orazio Zanetti, Daniel M. Portolani, João Marôco, Niels Janssen, Eva Y.L. Tan, Marjolein de Vugt, Frans Verhey, Manuel Gonçalves-Pereira

**Affiliations:** ^1^ NOVA National School of Public Health, Public Health Research Centre, NOVA University Lisbon, Lisbon, Portugal; ^2^ Comprehensive Health Research Centre (CHRC), LA-REAL, NOVA University Lisbon, Lisbon, Portugal; ^3^ Dementia Services Development Centre (DSDC) Wales, School of Medical and Health Sciences, Bangor University, Bangor, United Kingdom; ^4^ Department of Psychiatry and Neuropsychology and Alzheimer Centre Limburg, School for Mental Health and Neurosciences, Maastricht University, Maastricht, Netherlands; ^5^ School of Psychology, Dublin City University, Dublin, Ireland; ^6^ School of Nursing, Psychotherapy and Community Health, Dublin City University, Dublin, Ireland; ^7^ Institute for Health and Nursing Science, Martin Luther University Halle-Wittenberg, Halle (Saale), Germany; ^8^ Division of Neurogeriatrics, Department of Neurobiology, Care Sciences and Society, Karolinska Institutet, Stockholm, Sweden; ^9^ Norwegian National Centre for Ageing and Health, Vestfold Hospital Trust, Tønsberg, Department of Geriatric Medicine, Oslo University Hospital, Faculty of Medicine, University of Oslo, Oslo, Norway; ^10^ IRCCS Istituto Centro San Giovanni di Dio Fatebenefratelli, Brescia, Italy; ^11^ William James Center for Research, Instituto Superior de Psicologia Aplicada – Instituto Universitário (ISPA-IU), Lisboa, Portugal; ^12^ Institute for Mental Health Care, GGzE, Eindhoven, Netherlands; ^13^ Faculdade de Ciências Médicas, NOVA Medical School, Universidade NOVA de Lisboa, Lisbon, Portugal

**Keywords:** Alzheimer’s disease, close relationships, family care, informal care, latent profile analysis, longitudinal study, social support, quality-of-life

## Abstract

**Objective:**

The quality of the relationship between persons with dementia and family carers influences health and quality-of-life outcomes. Little is known regarding those at higher risk of experiencing a decline in relationship quality, who could potentially benefit the most from interventions. We aimed to identify these risk profiles and explore the underlying factors.

**Methods:**

We applied a latent profile analysis to relationship quality data from a 1-year follow-up of 350 dyads of persons with dementia and their informal carers from the Actifcare cohort in eight European countries. Assessments included sociodemographic, clinical, functional, psychosocial and quality-of-life measures. Relationship quality was assessed with the Positive Affect Index. A discriminant analysis explored factors influencing the risk profiles.

**Results:**

There were two relationship quality profiles among persons with dementia (gradually decreasing, 74.0%; low but improving, 26%) and two among carers (steadily poor, 57.7%; consistently positive, 42.3%). The ‘gradually decreasing’ profile (persons with dementia) was related to their levels of dependence and unmet needs, along with carers’ social distress and negative feelings, lower baseline RQ and sense of coherence. The ‘steadily poor’ profile (carers) was influenced by their social distress and negative feelings, lower sense of coherence and perceived social support. These two predominant profiles showed significant decreases in quality-of-life over one year.

**Conclusions:**

Specific profiles of persons with dementia and their carers are at risk of worse relationship quality trajectories. By considering modifiable related factors (e.g., carers’ stress), our findings can help develop tailored, effective interventions.

## Introduction

The presence and nature of close relationships may influence the onset and prognosis of several chronic health conditions, being robust predictors of quality and length of life (1). Among such health conditions, dementia has a heavy ‘burden of disease’ and impact on families and informal carers (2). Worldwide, around 55 million people live with dementia and this number is expected to reach 152.8 million in 2050 (3). Overall, families remain the cornerstone of care for older people, but they need support to persevere in their role as long as possible. It would be helpful if social and health care professionals were able to reliably identify people at risk of poor outcomes based on relationship factors.

In dementia, previous research provided support for the association of the relationship quality between the informal carer (i.e. family, friends) and the person with dementia, and a range of outcomes, including challenging behaviour (e.g., agitation, aggression, apathy), cognitive and functional decline, quality-of-life and institutionalisation (4–7). Carers who report poorer relationship quality are at greater risk of subjective burden and psychological distress, including anxiety and depression (8–10).

Most studies measure relationship quality at a single point, missing temporal changes. The few longitudinal studies of factors associated with poor relationship quality pathways highlighted the role of carers’ stress, depression and anxiety, and neuropsychiatric symptoms and self-reported quality-of-life of persons with dementia (11–13). Overall, these studies relied on relatively small convenience samples (12, 13), excluded the perspectives of persons with dementia (11), focused on young-onset dementia (13), or only examined spousal carers (11, 12). Moreover, they did not analyse profiles at higher risk of poor-quality relationships.

Meanwhile, several reviews called for more robust evidence regarding the role and course of relation quality (14). Particularly, studies on its association with neuropsychiatric symptoms are inconclusive, with findings ranging from no significant association (15) to identifying relationship quality as a target of intervention for less severe neuropsychiatric symptoms (8, 16).

Optimising aspects of the person with dementia-carer relationship could potentially improve outcomes in dementia (17), probably in more effective ways if we could selectively target those persons with dementia and carers at risk of worse relationship quality trajectories over time. To our knowledge, no study has explored the existence of profiles (subpopulations of individuals) differentiated according to this important variable and its influencing factors. To improve our ability to support these families, this evidence gap must be addressed.

In our previous cross-sectional study from a large European cohort (18) carer stress, expressed as anger and frustration, was the only factor associated with both person with dementia and carer perceptions of relationship quality, which frequently diverge. A stronger sense of coherence (SOC) in carers, i.e. a dispositional orientation toward a positive life (19), and being a spouse/partner (versus an adult child carer) were also related to their better perception of the relationship (18). Subsequently, we analysed the same Actifcare cohort regarding the trajectories of relationship quality among persons with dementia and found that their ratings did not decline significantly. In contrast, carer ratings showed a significant decline over time (20). Notably, our longitudinal and comprehensive data would allow us to delve deeper into the existence of relationship quality profiles and examine a broader range of influencing factors than those previously considered; these include demographics and type of relationship but also variables amenable to intervention, such as psychosocial unmet needs, neuropsychiatric symptoms, along with carers’ SOC, stress, depression and anxiety. Additionally, it would be interesting to analyse differences in quality-of-life between these profiles. As relationship quality is a key component of quality-of-life, increasing our understanding of the former, may provide us with a better picture of the many facets of the latter. Altogether, enlarging this evidence-base might shape early interventions targeting relationship quality-based risk profiles.

Therefore, in this paper we aim to: 1) identify distinct profiles based on relationship quality within a large cohort of persons with dementia and their informal carers followed over one year; 2) explore the factors influencing each profile; 3) further characterize the profiles, based on sociodemographic data and quality-of-life measures.

## Methods

### Participants

The data presented here are drawn from the 1-year prospective cohort Actifcare (ACcess to TImely Formal Care) EU-JPND project, a multimethod study in eight European countries: Germany, Ireland, Italy, Netherlands, Norway, Portugal, Sweden and United Kingdom. Actifcare comprised at least 50 dyads of community-dwellers with mild-to-moderate stages of dementia and their family carers per country, 451 in total.

Participants were recruited from various settings, including general practices, memory clinics and Alzheimer’s Associations. Persons with dementia were diagnosed according to the Diagnostic and Statistical Manual of Mental Disorders (DSM‐IV) criteria, staged mild to moderate using the Clinical Dementia Rating - CDR (21), with Mini Mental State Examination (MMSE) score of 24 or lower (22). Exclusion criteria included alcohol‐related dementia and receiving significant personal care from formal services (e.g., home care, day centres). For each person, their primary informal carer was selected. The full project protocol is detailed elsewhere (23).

### Procedures

Participants were assessed on entry (451 dyads), and again at approximately 6 (398 dyads) and 12 months (368 dyads). This paper reports on the 350 dyads that completed all three observations on an index of relationship quality, the Positive Affect Index (PAI) (Online **Appendix A**).

Participants were seen at home unless they preferred otherwise. Assessments were conducted separately with the person with dementia and their carer but, when necessary, part would take place conjointly (mainly if the person with dementia felt more secure in the presence of their loved one). Typically, each visit lasted up to two hours and was split into two sessions, if needed, to minimise evaluation overload. Trained staff conducted the comprehensive assessments.

Ethical approval was granted in each of the eight countries and all procedures followed the Helsinki Declaration of the World Medical Association. Persons with dementia and their carers provided informed written consent according to national regulations (23).

### Measures

Participants completed questionnaires on sociodemographic information (e.g., gender, age, education) and clinical-functional measures (23). Those selected for this study are outlined below.

The PAI (24) assessed current relationship quality, and was rated separately by persons with dementia and carers. Ratings of persons with dementia were used to define their profiles, and carers’ ratings to define their own’s. The PAI comprises five items, closeness, communication, similar views, shared activities, and generally getting along. Responses are rated on a 6-point scale from 1 (not well) to 6 (extremely well), with a total sum score from 5 to 30 (higher scores reflecting better RQ). This scale has been used with persons with dementia (12), showing good internal consistency (Cronbach α = 0.81) and reasonable test-retest reliability (*r* = .66) (25). In the present study, Cronbach’s αs were 0.82 (people with dementia) and 0.79 (carers).

Measures for persons with dementia included: CDR (21), MMSE (22), Neuropsychiatric Inventory Questionnaire (NPI-Q), with symptom count and separate scores for severity and carer distress (26), Instrumental Activities of Daily Living (IADL) and Physical Self-Maintenance Scale (PSMS) (27). The NPI-Q, IADL and PSMS were completed from carers’ reports. The Quality-of-Life-Alzheimer’s Disease (QOL-AD) (28) and the DEMQOL-U were used as disease-specific quality-of-life scales for use in mild to moderate dementia. Both were interviewer administered and their proxy-report versions were also used (29–31).

Carers’ assessments included the Hospital and Anxiety Depression Scale (HADS) (32) and the Relative Stress Scale (RSS) (33). In addition to RSS total scores, 3 sub-scores were calculated (emotional distress, social distress, negative feelings toward the person with dementia) (34). Carers’ QoL, perceived social network and SOC were measured with the CarerQol (35), the Lubben Social Network Scale (LSNS-6) (36), and the 13-item *Orientation to Life Questionnaire*, commonly known as the SOC scale (19). The Camberwell Assessment of Need (CANE) assessed 24 areas of need, covering biopsychosocial domains, with two additional items on carers’ own needs: information and psychological distress (37).

### Statistical analysis

LPA is a person-centred analytic tool that focuses on similarities and differences among people instead of relations among variables. It assesses whether the probability of belonging to a specific profile can be explained by individual characteristics, such as risk or protective factors (38). LPA utilized the intercept (initial relationship quality level) and slope (change rate) data from both persons with dementia and carers. Demographics and other covariates (e.g., ADL/IADL function, perceived social support) were employed to differentiate the profiles. To identify distinct profiles based on relationship quality, we performed a LPA in the slope and intercept data for both person with dementia and carers samples using the *mclust* R package (39). Variables with less than 10% missing data were imputed using Full Information Maximum Likelihood (FIML) methods.

Fit was judged from the *χ* ^2^ goodness of fit statistic, Akaike Information Criterion (*AIC*) and Bayesian Information Criterion (*BIC*). ‘The smaller the better’ rule was used to choose the best LPA model.

Linear discriminant analysis of profiles obtained for person with dementia and carers was performed with the package *MASS* (40) for the R Statistical System.

A stepwise forward selection procedure was used to identify the statistically significant predictors of the inclusion of people with dementia and carers into specific profiles for a p-level entry of 0.2 using the package *klaR* (41) for the R Statistical System.

To examine whether sociodemographic variables and quality-of-life were associated with the profiles we conducted independent‐sample t-tests and repeated measures ANOVA. The significance threshold was set at 0.05.

We used the Statistical Package for the Social Sciences (SPSS) for Windows version 26 and R Statistical System (v. 4.0) (42).

## Results

The characteristics of the total Actifcare cohort (451 dyads of persons with dementia and their carers) were previously reported (18, 43).

The demographic and clinical characteristics of participants who completed three PAI assessments (*n* = 350 dyads) are summarised in **Table 1**. This sample differed significantly from the remaining Actifcare dyads in persons with dementia’s lower age, dementia severity, level of neuropsychiatric symptoms and dependence, and their carers’ lower level of depressive symptoms and stress (**Table 1**). Attrition was due to the fact that participants were no longer willing to collaborate, stating exhaustion, health issues, moving abroad, institutionalisation or death (Online **Appendix A**).

**Table 1:** Baseline characteristics and summary of measures for those included and excluded from the analyses for both persons with dementia and carers.

	Included sample (*n* = 350)		Other Actifcare participants (*n* = 101)		
	*n* or mean	% or (SD)	*n* or mean	% or (SD)	Sig.
Persons with dementia					
Gender, women	189	54.3	57	55.3	.910
Age, years	77.06	(7.92) range 47-94	80.18	(7.13) range 49-95	.001***
Education, years	9.95	(4.53)	9.39	(4.26)	.271
Living alone	286	82.2	77	74.8	.119
Type of dementia					.105
Alzheimer’s Type	176	50.9	42	40.8	
Vascular	36	10.4	16	15.5	
Lewy Body	3	0.9	3	2.9	
Mixed (Alzheimer’s and Vascular)	38	11.0	18	17.5	
Not Known	71	20.5	19	18.4	
Other	22	6.4	5	4.9	
Cognitive impairment (MMSE)	19.11	(4.96)	18.64	(5.08)	.432
Dementia severity (CDR)	6.92	(2.36)	7.55	(2.59)	.023*
Neuropsychiatric symptoms (NPI-Q)	7.50	(5.39)	8.94	(5.87)	.026*
Severity (NPI-Q)	7.91	(5.18)	8.65	(5.95)	.231
IADL function (IADL)	3.57	(1.97)	3.00	(1.96)	.012*
Basic ADL function (PSMS)	3.81	(1.78)	3.09	(2.04)	.002**
Relationship quality (PAI)	22.85	(3.94)	22.72	(3.81)	.254
Quality-of-life (QoL-AD, self-rated)	36.12	(6.14)	35.93	(6.93)	.311
Carers					
Carer - Gender, women	230	66.3	69	67.0	1.000
Carer - Age, years	66.17	(13.20) range 47-94	67.19	(13.44) range 48-95	.490
Carer - Education, years	11.98	(4.48)	11.61	(4.20)	.457
Relationship to the person with dementia					.222
Spouse (wife/husband)	228	65.5	60	58.3	
Son/daughter	103	29.6	34	33.0	
Other	17	4.9	9	8.7	
Depression (HADS)	4.52	(3.60)	5.66	(3.60)	.005**
Anxiety (HADS)	6.06	(3.83)	6.56	(3.76)	.242
Distress (NPI-Q)	7.91	(5.18)	8.65	(5.95)	.231
Social support (LSNS-6)	16.61	(5.57)	16.55	(5.51)	.927
Stress (RSS)	20.57	(10.72)	23.83	(11.20)	.008**
Sense of coherence (SOC)	67.27	(10.79)	66.64	(11.52)	.612
Relationship quality (PAI)	21.31	(4.42)	21.24	(4.35)	.263
Quality-of-life (CarerQol-7D)	9.53	(2.56)	9.49	(2.45)	.109

CarerQol-7D, Carers’ quality-of-life, CDR, Clinical Dementia Rating Scale; HADS, Hospital Anxiety and Depression Scale; IADL, Instrumental Activities of Daily Living; LSNS, Lubben Social Network Scale; MMSE, Mini‐Mental State Examination; NPI‐Q, Neuropsychiatric Inventory Questionnaire; PAI, Relationship Quality; PSMS, Physical Self‐Maintenance Scale; QoL-AD, Quality of Life-Alzheimer’s Disease; RSS, Relative Stress Scale; SD, Standard Deviation; SOC, Sense of Coherence.

For the NPI, the carer rated the person with dementia symptoms, their severity, and the degree of distress. * p ≤ .05, ** p ≤ .01, *** p ≤ .001.

### Relationship quality profiles among persons with dementia and carers

Mean PAI scores, as rated by people with dementia, did not decline, considering the baseline (T0), 6 months (T1) and 12 months (T2) follow-up assessments (22.91; 22.52; 22.62). However, the differences in PAI assessments by carers across the three time points were are all statistically significant F(2 646) = 36.494, *p* = .001, with a decline over time (21.32; 20.75; 19.79).

No severe deviations from normality were observed in any PAI score (baseline, 6- and 12-month follow-up) (|Sk|<3 and |ku|<7) and thus Maximum Likelihood methods were appropriate for LPA.

The application of LPA resulted in two distinct profiles of relationship quality among persons with dementia (Online **Appendix B**). Profile 1 (*n* = 91, 26%) started with lower PAI and improved (‘low but improving’). The larger profile 2 (*n* = 259, 74%) started with higher PAI and decreased over time (‘gradually decreasing’) (Online **Appendix C**). Regarding carers, the best fit in Bayesian Information Criterion (BIC) yielded two profiles with clearly distinguishable relationship quality longitudinal dynamics (Online **Appendix D**). Profile 1 (‘consistently positive’) represented 42.3% (*n* = 148) of the carers. Profile 2 (‘steadily poor’) represented 57.7% (*n* = 202) (Online **Appendix E**).

### Factors influencing profile membership

Mean ratings for potential baseline predictors of persons with dementia and carers’ profile membership, at baseline and one-year follow-up, are displayed in **Tables 2**, **3**.

**Table 2:** Mean ratings for profiles among persons with dementia at baseline and follow-up.

	Persons with Dementia			
	Profile 1 ‘low but improving’ (*n*=91)		Profile 2 ‘gradually decreasing’ (*n*=259)	
	Baseline	Follow-up 2	Baseline	Follow-up 2
Variable	M	M	M	M
IADL function (IADL)	3.78	2.68	3.47	2.65
Basic ADL function (PSMS)	3.69	2.87	3.83	3.02
Neuropsychiatric symptoms (NPI-Q)	7.93	8.06	7.18	7.72
Anxiety (HADS)	6.52	6.87	5.91	6.45
Depression (HADS)	5.03	5.96	4.30	5.18
Emotional distress (RSS)	10.04	9.88	8.78	9.50
Social distress (RSS)	7.11	8.51	7.29	8.48
Negative feelings (RSS)	4.62	3.92	4.10	3.69
Social support of carer (LSNS)	16.06	15.24	16.83	16.45
Carer sense of coherence (SOC)	63.83	65.10	68.56	68.64
Person with dementia relationship quality (PAI)	19.68	20.59	23.93	23.35
Carer relationship quality (PAI)	19.52	18.52	21.93	20.24
Person with dementia unmet needs (CANE)	2.06	1.89	1.46	1.31

**Table 3:** Mean ratings for profiles among carers at baseline and follow-up 2.

	Carers			
	Profile 1 ‘consistently positive’ (*n*=148)		Profile 2 ‘steadily poor’ (*n*=202)	
	Baseline	Follow-up 2	Baseline	Follow-up 2
Variable	M	M	M	M
IADL function (IADL)	3.83	3.00	3.35	2.41
Basic ADL function (PSMS)	4.18	3.41	3.51	2.67
Neuropsychiatric symptoms (NPI-Q)	6.07	6.99	8.33	8.78
Anxiety (HADS)	4.95	5.48	6.90	7.3
Depression (HADS)	3.34	4.14	5.36	6.3
Emotional distress (RSS)	7.18	7.78	10.54	10.98
Social distress (RSS)	5.58	6.98	8.47	9.62
Negative feelings (RSS)	3.16	2.90	5.03	4.39
Social support of carer (LSNS)	17.48	17.20	16.01	15.34
Sense of coherence (SOC)	71.13	71.23	64.52	65.10
Person with dementia relationship quality (PAI)	23.39	23.05	22.45	22.20
Carer relationship quality (PAI)	23.14	22.19	19.97	18.01
Person with dementia unmet needs (CANE)	1.33	1.07	1.82	1.77

Persons with dementia’ profiles differed across basic ADL function (PSMS) at baseline, with the largest (standardized) regression coefficient (λ=0.302) followed by IADL function (λ=-0.261) and carers’ psychological distress unmet needs (λ=-0.209), with higher level of ADL function and lower levels of carer distress in profile 1 (‘consistently positive’) (Online **Appendix F**). Using a stepwise forward selection method, significant predictors of profile membership among persons with dementia, included RQ carer perspective F(1, 342) = 22.799; p<.001, IADL function F(1, 342) = 13.313; p<.001, basic ADL function F(1, 342) = 11.006; p<.001, carers’ SOC F(1, 342) = 8.914; p<.001, social distress F(1, 342) = 7.737; p<.001 and negative feelings F(1, 342) = 22.799; p<.001 and persons with dementia’ unmet needs F(1, 342) = 6.594; p<.001 (**Table 4**).

**Table 4:** Summary of the stepwise regression analysis for the baseline variables influencing profile membership among people with dementia.

Variable	Wilks’ Lambda	F	df1	df2	Sig.
Carer relationship quality (PAI)	.931	22.799	1	342	<.001
IADL function (IADL)	.920	13.313	1	342	<.001
Basic ADL function (PSMS)	.903	11.006	1	342	<.001
Carer sense of coherence (SOC)	.896	8.914	1	342	<.001
Social distress (RSS)	.888	7.737	1	342	<.001
Negative feelings (RSS)	.876	7.229	1	342	<.001
Person with dementia unmet needs (CANE)	.868	6.594	1	342	<.001

The discriminant analysis with a stepwise procedure allows to select only the important baseline variables that influence profile membership, while redundant variables (variables that contribute less in the presence of other variables) are discarded. CANE, Camberwell Assessment of Need for the Elderly; IADL, Instrumental Activities of Daily Living; PAI, Positive Affect Index; PSMS, Physical Self‐Maintenance Scale; RSS, Relative Stress Scale; SOC, sense of coherence.

Regarding carers, variables contributing the most to discriminate the ‘consistently positive’ and ‘steadily poor’ profiles included carer depression (λ=0.294), type of relationship (e.g., spouse/partner, adult child) (λ=0.291), and RSS ‘negative feelings’(λ=-0.209) (Online **Appendix G**). Stepwise discriminant analysis identified the most important baseline predictors of carers’ profiles, including RSS ‘negative feelings’ F(1, 345) = 36.899; p<.001, ‘social distress’ F(1, 345) = 6.594; p<.001, SOC F(1, 345) = 24.986; p<.001, and perceived social support F(1, 345) = 18.147; p<.001 (**Table 5**).

**Table 5:** Summary of the stepwise regression analysis for the baseline variables influencing profile membership among carers.

Variable	Wilks’ Lambda	F	df1	df2	Sig.
Negative feelings (RSS)	.894	36.899	1	345	<.001
Carer sense of coherence (SOC)	.896	24.986	1	345	<.001
Social support of carer (LSNS)	.851	18.147	1	345	<.001
Social distress (RSS)	.868	6.594	1	345	<.001

The discriminant analysis with a stepwise procedure allows to select only the important baseline variables that influence profile membership, while redundant variables (variables that contribute less in the presence of other variables) are discarded. LSNS, Lubben Social Network Scale; RSS, Relative Stress Scale; SOC, sense of coherence.

### Differences between profiles on sociodemographic and quality-of-life variables

Finally, we further characterised the profiles based on baseline sociodemographic characteristics (age, gender, and education) and QoL measures (QoL-AD, DEMQoL-U and CarerQol) across T0, T1 and T2.

No significant differences were found for age, gender, or education within any profile.

When analysing each ‘persons with dementia’ profile for quality-of-life measures, we found significant differences on QoL-AD (self-rated) scores in the largest profile (74%) ‘Relationship quality gradually decreasing’ F(2, 170) = 5.771, *p* = .004, between T0 and T2 (*p* = .003), with a higher mean at T0 (36.72 vs 35.62). In the same profile, there were differences regarding QoL-AD (carer’s proxy ratings) scores F(2, 188) = 6.473, *p* = .002, between T0 and T2 (*p* = .002) and T1 and T2 (*p* = .025).

Analysing the other ‘persons with dementia’ profile (‘low but improving’) there were significant differences on DEMQoL-U (proxy) F(2, 51) = 3.975, p = .025, between T0 and T2 (*p* = .019), with a higher mean at T2. The two profiles differed on the DEMQoL-U proxy (p = .001) and CarerQoL (p = .003) baseline scores.

When analysing each ‘carer’ profile, we found significant differences on QoL-AD (carer’s proxy ratings) scores in the profile ‘steadily poor’ F(2, 164) = 6.121, *p* = .003. From T0 to T2 (*p* = .003), the average score decreased (36.17 vs 34.91). The same applied to QoL-AD (self-rated) scores F(2, 185) = 4.261, *p* = .016, with a lower mean at T2 (30.71 vs 29.58).

The two ‘carer’ profiles (‘steadily poor’, ‘low but improving’) differed on baseline QoL-AD (self-rated) (p = .001), DEMQoL proxy (p = .012) and CarerQoL (p = .012) scores.

## Discussion

We identified distinct profiles based on relationship quality, in a large cohort of European community-dwellers with dementia and their family carers, followed over one year. Using a novel statistical approach, our analyses focused on identifying subgroups at risk of poor related outcomes. There were two main profiles, both characterized by relationship quality decline over time: the ‘gradually decreasing’ profile (representing 74% of persons with dementia) and the ‘steadily poor’ profile (corresponding to 58% of their carers). Carer’s stress and SOC emerged as common factors explaining profiles of both persons with dementia and carers at higher risk of decline. We also documented expectable links between carer relationship quality and quality-of-life in dementia. Overall, our objectives were met: first, to identify distinct profiles based on relationship quality; second, to explore the factors influencing each profile; and third, to further characterise the profiles using demographic data and quality-of-life measures.

### Profiles of persons with dementia

Using LPA proved useful to identify specific profiles that showed distinct PAI changes over time (‘gradually decreasing’; ‘slow but improving’). The larger profile of persons with dementia (74%), whose relationship quality declined over time (‘gradually decreasing’), was determined by a mix of carer and person with dementia characteristics. These included, from the carer’s perspective, lower PAI, lower SOC, and higher stress, together with reduced function and more unmet needs of the person with dementia.

Regarding the influencing factors, relationship quality (as rated by the carer) added to the prediction of persons with dementia profiles. Since relationships are based on reciprocity, it is reasonable to assume that the person with dementia’s perspective is influenced by the carer’s attitudes (‘partner effect’) (44). This finding supports the value of considering both perspectives within the dyad.

Present findings extend the evidence by supporting lower carer’s SOC as an indicator to help identifying subgroups of persons with dementia at risk. Previously, we had shown how carer’s SOC may protect relationships (18).

In line with previous research, carers’ distress emerged as one of the strongest predictors of relationship quality profiles in persons with dementia. The at-risk profile seemed especially related to carer’s social distress (i.e. feelings of being limited in terms of social life) and negative feelings towards the care-receiver (e.g., anger, frustration), suggesting responsiveness from the person with dementia to the family’s emotional climate (7).

Reduced functional abilities of the person with dementia also influenced inclusion in the vulnerable profile. Perhaps less functional abilities make them more prone to assess relationships negatively, creating imbalances through increased dependence of carers. Alternatively, feeling overwhelmed contributed to carers’ negative appraisals of persons with dementia’ function, as IADL and PSMS are assessed by them.

To our best knowledge, this is the first study to underline links between unmet needs and lower relationship quality in dementia, which was not surprising. Indeed, the most reported unmet need in the Actifcare cohort was ‘company’ (43), a salient unmet need in community-dwellers with dementia (45).

Finally, we characterised the profiles using demographics and quality-of-life measures. Demographics were not associated with any probability of belonging to a specific profile. The profile with a decrease of PAI (‘gradually decreasing’) was also marked by a decline in quality-of-life during the follow-up (QoL-AD and DEMQoL-U scores). Our characterisation of the two profiles of persons with dementia, based on self-report, disease-specific quality-of-life measures (QoL-AD and DEMQoL), enabled us to hear the care-receiver’s perspective first-hand and highlighted the value of focusing on relationship factors to better understand quality-of-life in dementia. Preserving relationship quality proved to be an important key aspect of quality-of-life. As reported for the QoL-AD, persons with mild-to-moderate dementia can reliably appraise their relationships, and their perspective on this specific topic contributes to their self-reported quality-of-life overall (46, 47). This finding contributes to filling gaps in knowledge on how relationship factors impact on persons with dementia quality-of-life, as requested by a recent review (14).

### Profiles of carers

The two carer profiles were ‘steadily poor’ and ‘consistently positive’ relationship quality. The larger profile (‘steadily poor’) was determined by carer characteristics only (RSS ‘negative feelings’ and ‘social distress’, SOC and perceived social support). Using LPA enabled us to identify another profile of carers that maintained positive PAI scores (‘consistently positive’) despite adversity and facilitated our exploration of factors contributing to more positive experiences.

Regarding the factors influencing the profiles, and in line with our previous results (18), carers’ negative feelings (e.g., anger, frustration) were significantly associated with lower PAI. Regarding ‘social distress’, one possible interpretation is that, as dementia progresses, the person with dementia becomes more dependent on their carer; with time, this often leads to restrictions on the carer’s social life, increasing feelings of isolation and loss of control.

As expected, their SOC was among the determinants of carers’ relationship quality profiles. A higher SOC predisposes carers to positively reframe or compensate for negative life events, potentially impacting on how relationships are appraised. We also cannot exclude a bidirectional interaction between SOC and relationship quality: poor quality could arguably contribute to lower SOC self-appraisals at a given point.

Also as expected, lower levels of perceived support were associated with more negative relationship quality assessments over time.

The more in-depth comparison of carers’ profiles based on demographics and quality-of-life measures helped us to complete the circle. There were no significant differences found among carer profiles for age, gender and education. Finally, just as relationship quality has a major influence on the quality-of-life of the person with dementia, it is also crucial to the carer’s (48), providing insight on the role of relationships as a component of quality-of-life in dementia. When analysing the ‘steadily poor’ carer’s profile, we found a deterioration of QoL-AD (both on carer’s ratings and care-receiver’s self-reports) over one year. This interestingly underlines that person with dementia quality-of-life self-reports also impact on carer’s relationship quality perspective. Moreover, there were differences between the two carer profiles regarding DEMQoL and CarerQol, as between the two profiles of persons with dementia. In the case of carer profiles, there were also differences on the QoL-AD (person with dementia self-rated). Consistent with reports that higher quality-of-life of the person with dementia was associated with higher relationship quality (both following carers’ perspectives) (13), our study further unveils some links between self-reported quality-of-life of the person with dementia and the carer’s relationship perspective.

### Strengths and limitations

The strength of the study lies in the fact that it is one of the very first to use a longitudinal design with repeated assessments to analyse relationship quality, rather than aiming only at a snapshot as in most previous cross-sectional studies. Our work goes beyond the previous literature by considering both persons with dementia and their family carers’ perspectives in a large cohort from different countries in Europe. Moreover, we used a novel statistical approach (LPA), appropriate to identify the profiles.

There are also limitations to acknowledge. First, the sample may not be representative, limiting generalizability. Attrition inevitably led to some degree of selection bias, with those lost to follow-up likely to be older and more severely impaired, and their carers reporting higher levels of depression symptoms and stress. Second, a longer follow-up than our 12 month-period could have resulted in different profiles and identified influencing factors. Finally, we did not consider the influence of a variety of potentially important factors, including the subtype of dementia (although we did consider neuropsychiatric symptoms), the receipt of formal care (in fact, an exclusion criterion at baseline), or time assisting with activities of daily living (analysed in our baseline study of relationship quality (18), but excluded here to achieve a model that strikes a balance between fit and complexity. Relationship quality prior to the onset of dementia obviously influences current relationship appraisals but its retrospective assessment would be heavily prone to bias in this research context.

## Conclusions

There is no one-size-fits-all solution to predict how relationship quality will change over time. However, specific factors like carers’ stress and social support are amenable to intervention and may help to identify profiles of persons with dementia or their family carers at risk of worse trajectories. These are the individuals that could benefit the most from timely psychosocial interventions which, by considering relationship quality, turn out to be intrinsically systemic. Our findings show that addressing aspects of the relationship quality may potentially improve health and quality-of-life outcomes in dementia.

## Data Availability

The datasets presented in this article are not readily available due to privacy restrictions on personal data. Requests to access the datasets should be directed to the corresponding author.
