# Supplementary material for: Addressing relationship quality of people with dementia and their family carers: which profiles require most support?
Source: Front Psychiatry. 2024 Sep 11;15:1394665. doi: 10.3389/fpsyt.2024.1394665 (PMC11422148; doi:10.3389/fpsyt.2024.1394665)
Supplement: Supplementary file 1 [file DataSheet1.pdf]

SUPPLEMENTARY MATERIAL

APPENDIX A. Flowchart of the dyads (persons with dementia and carers) included in the analysis from the Actifcare study

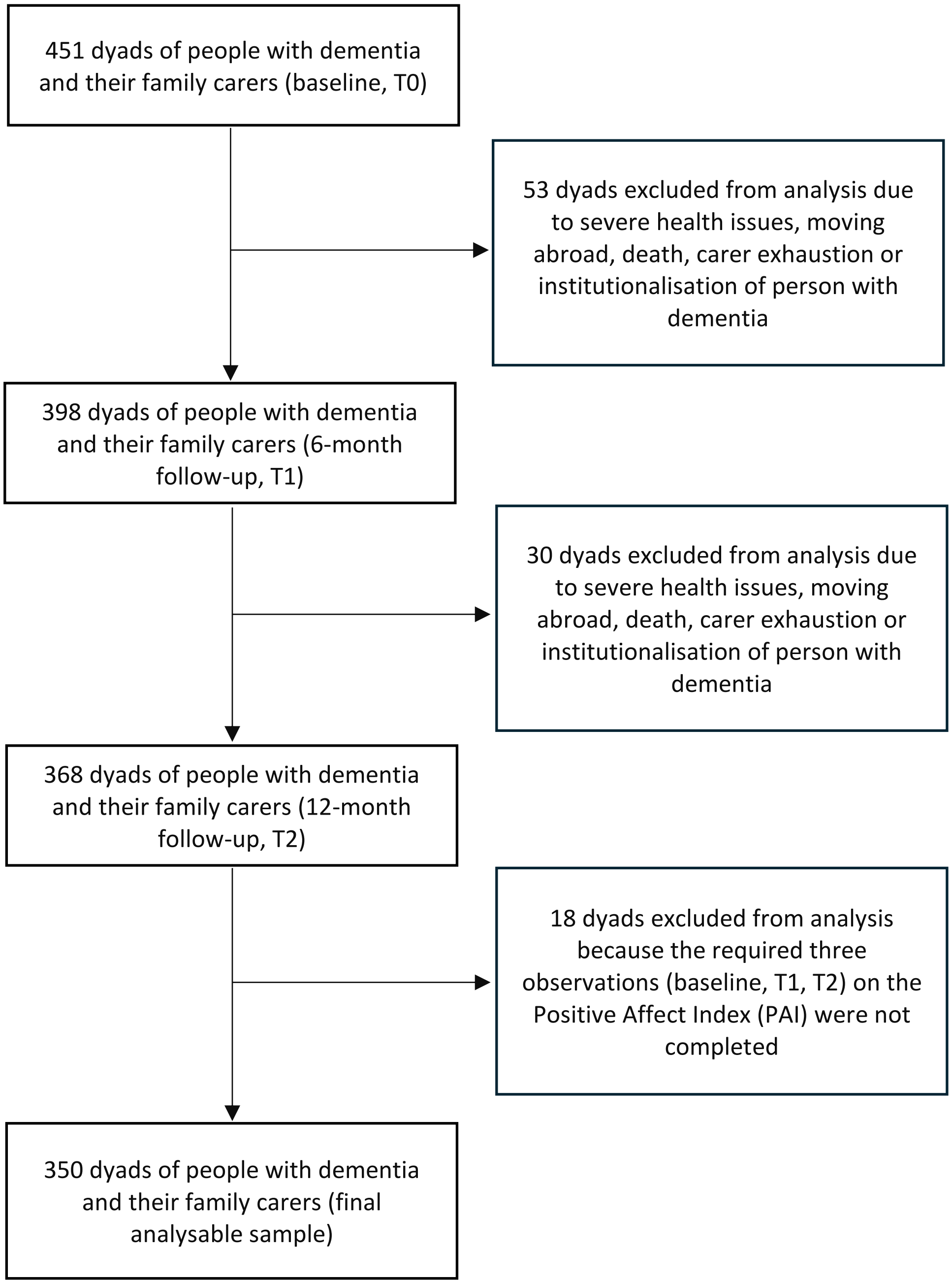

APPENDIX B. Elbow plot of the information criteria for the latent profile analysis among persons with dementia

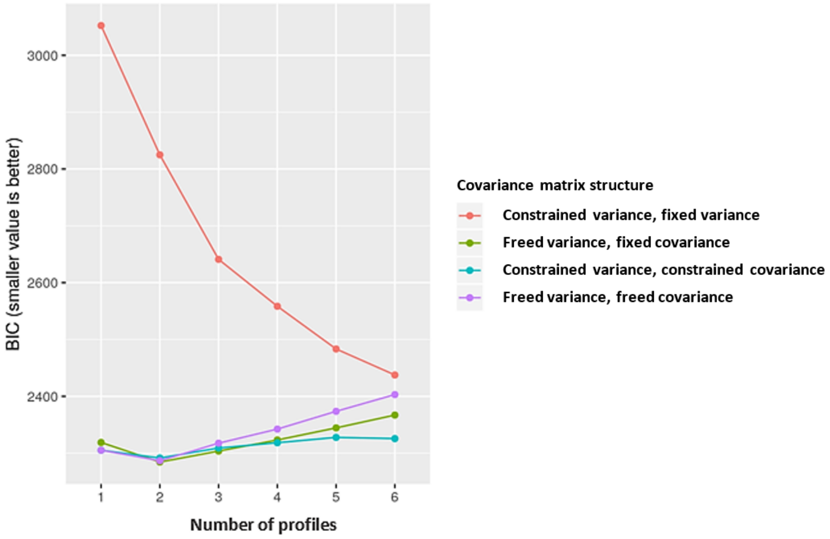

The elbow plot suggests that the improvement in fit reaches a plateau at two profiles, the model with the lowest Bayesian Information Criterion (BIC). According to the BIC, the best model is the one with freed variance and freed covariance with two latent profiles.

APPENDIX C. Bar chart modeling the two relationship quality profiles among persons with dementia

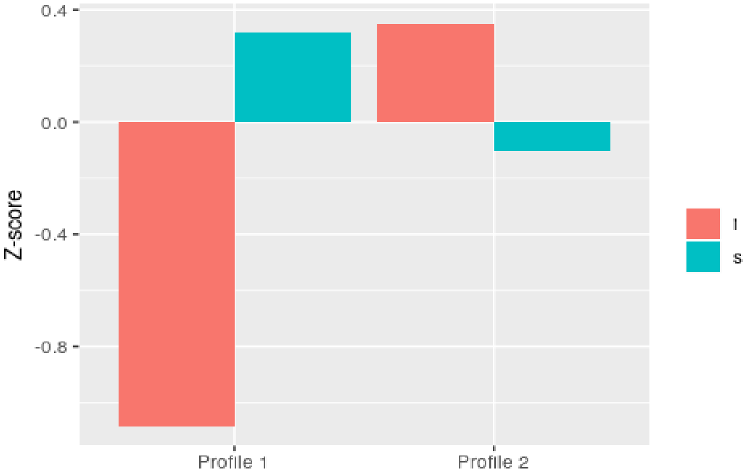

A positive Z-score indicates that the data point is above the mean (to the right on the normal distribution curve), while a negative Z-score indicates it is below the mean (to the left). A Z-score of 0 means the data point is exactly at the mean. The bar chart presents the two distinct profiles of relationship quality among persons with dementia. Profile 1 (‘low but improving’; *n* = 91, 26%) started with lower relationship quality (intercept) and improved over 1-year (slope). The larger profile 2 (‘gradually decreasing’; *n* = 259, 74%) started with higher relationship quality (intercept) and decreased over 1-year (slope).

APPENDIX D. Elbow plot of the information criteria for the latent profile analysis among carers

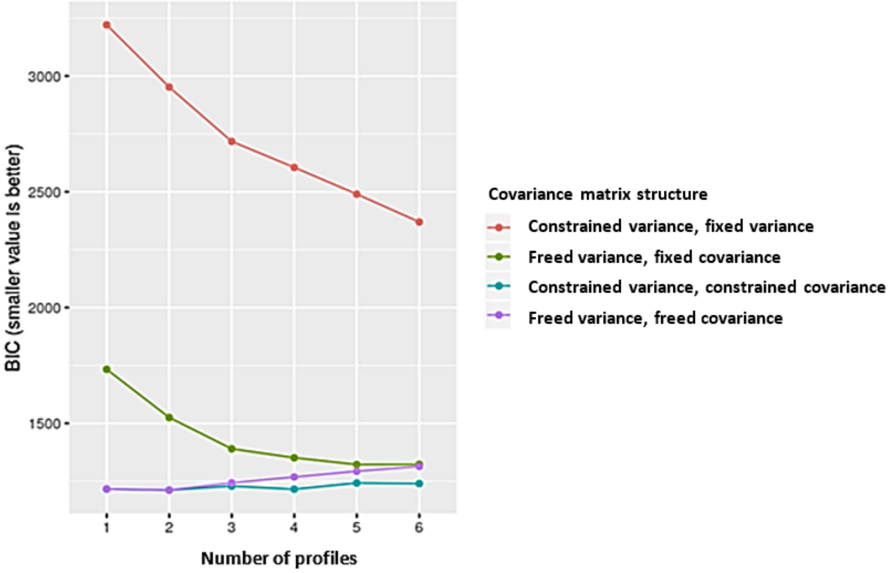

The elbow plot suggests that the improvement in fit reaches a plateau at two profiles, the model with the lowest Bayesian Information Criterion (BIC). According to the BIC, the best model is the one with freed variance and freed covariance with two latent profiles.

APPENDIX E. Bar chart modeling the two relationship quality profiles among carers

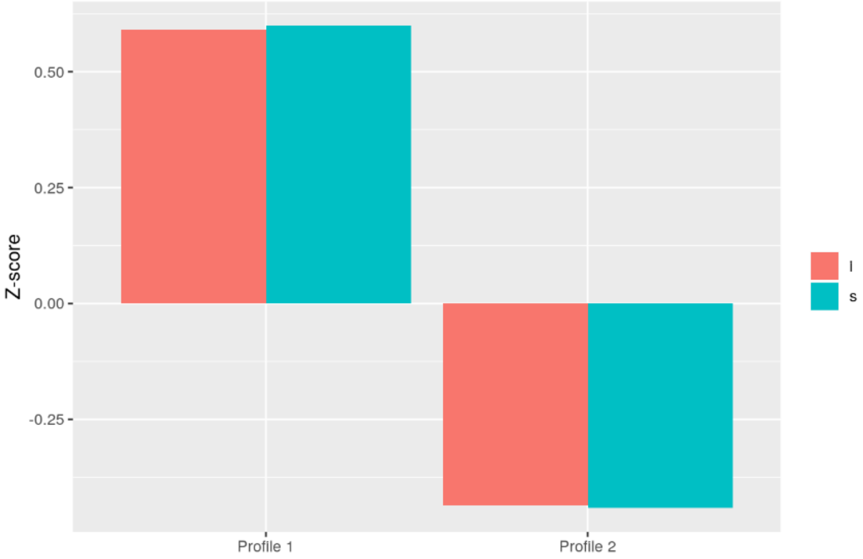

A positive Z-score indicates that the data point is above the mean (to the right on the normal distribution curve), while a negative Z-score indicates it is below the mean (to the left). A Z-score of 0 means the data point is exactly at the mean. The bar chart presents the two distinct profiles of relationship quality among carers. Profile 1 (‘consistently positive’; *n* = 148, 42.3%) maintained a positive relationship quality assessment over 1-year. Profile 2 (‘steadily poor’; *n* = 202, 57.7%) perceived more negatively relationship quality over the same period.

APPENDIX F. Coefficients of linear discriminants of persons with dementia' profiles

**Variable**	λ
IADL function (IADL)	-0.261
Carer psychological distress unmet needs (CANE)	-0.209
Person with dementia unmet needs (CANE)	-0.139
Emotional distress (RSS)	-0.100
Neuropsychiatric symptoms (NPI-Q)	-0.076
Spouse/partner relationship to the PwD	-0.066
Depression (HADS)	-0.031
Social support of carer (LSNS)	-0.022
Negative feelings (RSS)	0.013
Carer sense of coherence (SOC)	0.023
Anxiety (HADS)	0.032
Distress (NPI-Q)	0.059
Social distress (RSS)	0.128
Carer relationship quality (PAI)	0.129
Basic ADL function (PSMS)	0.301

Appendix E displays the coefficients or functions generated by the linear discriminant analysis that allow to identify the baseline variables that best differentiate (or discriminate) between the two different and mutually exclusive people with dementia' profiles. Abbreviations: HADS, Hospital Anxiety and Depression Scale; IADL, Instrumental Activities of Daily Living; LSNS, Lubben Social Network Scale; NPI-Q, Neuropsychiatric Inventory Questionnaire; PAI, Positive Affect Index; PSMS, Physical Self-Maintenance Scale; RSS, Relative Stress Scale.

APPENDIX G. Coefficients of Linear Discriminants of carers' profiles

**Variable**	λ
Carer psychological distress unmet needs (CANE)	-.065
Social support of carer (LSNS)	-.051
Carer sense of coherence (SOC)	-.034
Person with dementia relationship quality (PAI)	-.028
Person with dementia unmet needs (CANE)	-.012
Anxiety (HADS)	-.007
Distress (NPI-Q)	.001
Neuropsychiatric symptoms (NPI-Q)	.019
Emotional distress (RSS)	.025
Social distress (RSS)	.030
Negative feelings (RSS)	.166
Spouse/partner relationship to the PwD	.291
Depression (HADS)	.294

Appendix F displays the coefficients or functions generated by the linear discriminant analysis that allow to identify the baseline variables that best differentiate (or discriminate) between the two different and mutually exclusive carers' profiles. Abbreviations: CANE, Camberwell Assessment of Need for the Elderly; HADS, Hospital Anxiety and Depression Scale; LSNS, Lubben Social Network Scale; NPI-Q, Neuropsychiatric Inventory Questionnaire; PAI, Positive Affect Index; Relative Stress Scale; SOC, sense of coherence.
